# Supplementary material for: Identification of Early Biomarkers of Mortality in COVID-19 Hospitalized Patients: A LASSO-Based Cox and Logistic Approach
Source: Viruses. 2025 Feb 28;17(3):359. doi: 10.3390/v17030359 (PMC11946718; doi:10.3390/v17030359)
Supplement: Supplementary file 1 [file viruses-17-00359-s001.zip › viruses-3383621-supplementary.pdf]

Table S1. Overview of the amount of missing clinical data.

Feature	Nr. Missing Values
IL-10	1
IL-22	1
IL-6	1
IL-8	1
Transferrin	1
Transferrin sat.	6
Hb	1
CRP	8
RBC	1
WBC	1
Neutrophils	2
Lymphocytes	3
Monocytes	16
Platelets	1
AST	19
ALT	13
RDW	1
Hematocrit	14
PaO2/FiO2	2

The number of missing values for each feature with missing values.

Table S2. Wilcoxon test.

Feature	*p*-Value
LDH	3×10^{-5}
P/F	8×10^{-5}
IL-8	0.00446
eGFR	0.01248
IL-10	0.01692
Monocytes	0.02556
Lymphocytes	0.02846
IL-1β	0.04271
Creatinine	0.05485
IL-6	0.07273
Platelets	0.08262
TNF-α	0.09405
RDW	0.11863
IL-1Ra	0.25003
AST	0.26479
CRP	0.4489
Ferritin	0.64762
IL-22	0.69087
Hb	0.77603
Iron (Fe)	0.7765
ALT	0.78231
Hepc	0.82208
Neutrophils	0.86112
TF sat.	0.88366
WBC	0.88693
Hematocrit	0.9331
IFNγ	0.9348
RBC	0.96871
TF (transf)	0.99609

Wilcoxon test results comparing demographic, clinical, and biochemical features between survivor and non-survivor patient groups. *p*-values indicate the statistical significance of differences between the groups, with values <0.05 considered significant.

Table S3. Spearman significant correlation.

Feature	Feature	Correlation	*p*-Value
Hb	Hematocrit	0.9585	0.000
RBC	Hematocrit	0.9312	0.000
WBC	Neutrophils	0.9212	0.000
Hb	RBC	0.8809	0.000
Iron (Fe)	TF sat.	0.7495	1.577×10^{-14}
IL-1Ra	IL-6	0.7006	6.581×10^{-13}
WBC	Monocytes	0.6173	5.532×10^{-8}
IL-10	LDH	0.6066	3.128×10^{-9}
Ferritin	TF sat.	0.5446	5.288×10^{-7}
Neutrophils	Monocytes	0.5354	5.162×10^{-6}
IFNγ	IL-10	0.5233	7.507×10^{-7}
Lymphocytes	Monocytes	0.5174	1.196×10^{-5}
IL-1β	Lymphocytes	0.5082	2.380×10^{-6}
IL-8	TNF-α	0.5016	2.488×10^{-6}
Hepc	Ferritin	0.4752	8.439×10^{-6}
IL-6	CRP	0.4697	3.586×10^{-5}
IL-22	TNF-α	0.4679	1.374×10^{-5}
Monocytes	Platelets	0.4598	1.321×10^{-4}
IL-10	IL-6	0.4576	2.232×10^{-5}
IL-6	IL-8	0.4547	2.882×10^{-5}
Neutrophils	Platelets	0.4469	4.105×10^{-5}
WBC	Platelets	0.4443	4.093×10^{-5}
IL-10	IL-1Ra	0.4362	5.868×10^{-5}
Hepc	CRP	0.4361	1.287×10^{-4}
IL-1Ra	TNF-α	0.4305	6.724×10^{-5}
Age	IL-8	0.4232	1.018×10^{-4}
TNF-α	RDW	0.4200	1.165×10^{-4}
IL-6	TNF-α	0.4092	1.806×10^{-4}
IL-1Ra	IL-8	0.4041	2.216×10^{-4}
Age	TNF-α	0.4027	2.131×10^{-4}
IL-1β	P/F	0.3823	5.522×10^{-4}
Lymphocytes	P/F	0.3777	8.358×10^{-4}
IL-1Ra	CRP	0.3723	1.278×10^{-3}
TNF-α	Creatinine	0.3721	6.766×10^{-4}
IFNγ	LDH	0.3614	9.903×10^{-4}
Hepc	IL-6	0.3559	1.284×10^{-3}
IL-10	AST	0.3545	5.056×10^{-3}
IL-6	LDH	0.3458	1.802×10^{-3}

IL-1Ra	LDH	0.3405	1.995×10^{-3}
Hepc	LDH	0.3333	2.516×10^{-3}
Hepc	IFNγ	0.3268	3.091×10^{-3}
CRP	LDH	0.3228	5.676×10^{-3}
IL-10	TNF-α	0.3201	4.032×10^{-3}
Age	Creatinine	0.3053	5.892×10^{-3}
IL-8	RDW	0.3015	7.297×10^{-3}
IL-1Ra	Neutrophils	0.3001	7.592×10^{-3}
IFNγ	CRP	0.3001	1.044×10^{-2}
IL-1β	Monocytes	0.2856	2.215×10^{-2}
WBC	ALT	0.2827	2.043×10^{-2}
Neutrophils	ALT	0.2808	2.136×10^{-2}
IL-22	Creatinine	0.2796	1.258×10^{-2}
Age	IL-22	0.2707	1.583×10^{-2}
IFNγ	IL-1Ra	0.2673	1.655×10^{-2}
IL-1Ra	Monocytes	0.2663	3.344×10^{-2}
IL-1Ra	IL-22	0.2650	1.826×10^{-2}
WBC	Lymphocytes	0.2643	2.019×10^{-2}
IFNγ	IL-6	0.2640	1.872×10^{-2}
Iron (Fe)	WBC	0.2625	1.942×10^{-2}
IL-1β	TNF-α	0.2613	1.922×10^{-2}
TF sat.	Creatinine	0.2534	2.934×10^{-2}
IL-10	CRP	0.2514	3.446×10^{-2}
Hepc	Neutrophils	0.2498	2.741×10^{-2}
IL-1β	WBC	0.2494	2.664×10^{-2}
IL-8	Creatinine	0.2491	2.683×10^{-2}
Iron (Fe)	Neutrophils	0.2482	2.846×10^{-2}
Ferritin	CRP	0.2426	4.003×10^{-2}
IL-1β	IL-6	0.2407	3.260×10^{-2}
IL-10	IL-8	0.2403	3.406×10^{-2}
TF sat.	Hb	0.2391	4.166×10^{-2}
IFNγ	IL-22	0.2384	3.437×10^{-2}
Age	RDW	0.2377	3.491×10^{-2}
TF (transf)	Platelets	0.2351	3.826×10^{-2}
Platelets	eGFR	0.2350	3.713×10^{-2}
IL-1Ra	WBC	0.2324	3.928×10^{-2}
IL-22	IL-8	0.2320	4.098×10^{-2}
Iron (Fe)	Creatinine	0.2316	3.870×10^{-2}
IL-1Ra	IL-1β	0.2312	3.907×10^{-2}
Iron (Fe)	Hb	0.2272	4.409×10^{-2}
Hepc	IL-1Ra	0.2218	4.799×10^{-2}
TNF-α	Platelets	−0.2289	4.243×10^{-2}

eGFR	RDW	−0.2367	3.573×10^{-2}
IL-8	Hb	−0.2399	3.438×10^{-2}
IL-22	Platelets	−0.2444	3.107×10^{-2}
CRP	P/F	−0.2448	4.111×10^{-2}
CRP	Lymphocytes	−0.2525	3.362×10^{-2}
IL-6	Iron (Fe)	−0.2548	2.344×10^{-2}
IL-1Ra	TF sat.	−0.2598	2.538×10^{-2}
IL-1Ra	Hematocrit	−0.2679	2.965×10^{-2}
IL-8	Hematocrit	−0.2689	3.034×10^{-2}
CRP	Monocytes	−0.2691	4.105×10^{-2}
WBC	AST	−0.2736	3.290×10^{-2}
IL-1Ra	Hb	−0.2755	1.398×10^{-2}
IL-1β	LDH	−0.2760	1.321×10^{-2}
Iron (Fe)	AST	−0.2798	2.897×10^{-2}
Neutrophils	AST	−0.2841	2.651×10^{-2}
Iron (Fe)	LDH	−0.2882	9.517×10^{-3}
Platelets	AST	−0.2914	2.271×10^{-2}
IL-1Ra	Iron (Fe)	−0.2943	8.058×10^{-3}
TF sat.	AST	−0.2953	2.572×10^{-2}
IL-10	TF sat.	−0.3073	8.185×10^{-3}
IL-10	Iron (Fe)	−0.3087	5.645×10^{-3}
IL-22	eGFR	−0.3255	3.423×10^{-3}
Iron (Fe)	CRP	−0.3275	4.984×10^{-3}
IL-8	Platelets	−0.3380	2.477×10^{-3}
IL-8	eGFR	−0.3454	1.821×10^{-3}
TF (transf)	CRP	−0.3562	2.299×10^{-3}
Lymphocytes	LDH	−0.3633	1.164×10^{-3}
IFNγ	Iron (Fe)	−0.3778	5.501×10^{-4}
Hepc	RDW	−0.3924	3.485×10^{-4}
TNF-α	eGFR	−0.4012	2.258×10^{-4}
Hepc	TF (transf)	−0.4368	5.695×10^{-5}
Age	eGFR	−0.5155	9.917×10^{-7}
TF (transf)	TF sat.	−0.5452	5.086×10^{-7}
Ferritin	TF (transf)	−0.6414	1.908×10^{-10}
LDH	P/F	−0.7929	0.000
Creatinine	eGFR	−0.8908	0.000

The Wilcoxon test was employed to assess the statistical significance of differences between the groups of survivor and non-survivor, with a significance level of 5% ($p<0.05$).

Table S4. Schoenfeld residual test for proportional hazards assumption.

Marker	Chi-Squared	Degree of Freedom	*p*-Value
LDH	0.30827	1	0.579
IL-10	2.78771	1	0.095
eGFR	0.84778	1	0.357
Creatinine	1.15998	1	0.281
IFNγ	3.27219	1	0.07
Monocyte	0.55116	1	0.458
IL-22	1.74884	1	0.186
Hepcidine	0.00614	1	0.938
Hb	0.78373	1	0.376
IL-6	0.09509	1	0.758
Lymphocytes	1.46268	1	0.227
Age	2.27889	1	0.131
Global	11.23781	12	0.509

Schoenfeld residual test results for variables included in the final Cox proportional hazards model. The test assesses whether the proportional hazards assumption holds for each covariate and globally. A p-value > 0.05 indicates no significant violation of the assumption, confirming the appropriateness of the Cox model.
